# BMI modified the association of current smoking with the incidence of hypertension in Chinese population: a 22-year cohort study

**DOI:** 10.1186/s12889-020-8428-z

**Published:** 2020-03-06

**Authors:** Feifei Yao, Wenfeng Liu, Rencheng Zhao, Guangxiao Li, Xiaojuan Huang, Yongjie Chen

**Affiliations:** 1 The office of the top tertiary hospital, Shekou People’s Hospital, Nanshan district, Shenzhen, Guangdong Province China; 2 Center for Disease Control and Prevention of Changshan County, Quzhou, Zhejiang Province China; 3 Department of Chronic Non-communicable Diseases, Baoan Chronic Diseases Prevent and Cure Hospital, Shenzhen, Guangdong Province China; 4 The Medical Record Center of the First Affiliated Hospital of China Medical University, Shenyang, Liaoning Province China; 5 Health Inspection Institute of Changshan County, Quzhou, Zhejiang Province China; 6 Department of Epidemiology and Statistics, School of Public Health, Tianjin Medical University, 22 Qixiangtai Road, Tianjin, China; 7 Tianjin Key Laboratory of Environment, Nutrition and Public Health, 22 Qixiangtai Road, Tianjin, China

**Keywords:** The interaction effect, Obesity, Current smoking, The incidence of hypertension

## Abstract

**Background:**

There was little known on how the interaction effect between obesity and current smoking affected the incidence of hypertension. The aim of this study was to investigate how body mass index (BMI) modified the effect of current smoking on the incidence of hypertension.

**Methods:**

Data were obtained from the China Health and Nutrition Survey (CHNS). According to the WHO recommendations for Chinese people, the normal weight, overweight, and obesity were defined using the BMI cutoff values 18.5 kg/m^2^, 23.0 kg/m^2^, and 27.5 kg/m, respectively. Current smokers were defined as having smoked at least 100 cigarettes or electronic cigarettes, 20 cigars, or 20 tobacco pipes and other type of tobacco in the last 30 days preceding the survey. Hypertension was defined as systolic blood pressure (SBP)/ diastolic blood pressure (DBP) ≥ 140/90 mmHg, use of anti-hypertensive medications, or a self-reported diagnosis.

**Results:**

This study included 12,900 subjects. There were interaction effects between obesity and current smoking in females (*P* = 0.030) and the 50–59 years group (*P* = 0.049). Current smoking was a significant predictor of incident hypertension only in the total and female populations with normal weight (*HR*: 1.119 and 1.274; *HR* 95% *CI*: 1.013–1.236 and 1.143–1.415; and *P* = 0.027 and 0.040, respectively). Stratified by age, current smoking affected the development of hypertension only in the 50–59 years subjects with the normal weight (*HR*: 1.356; *HR* 95% *CI*: 1.084–1.697; and *P* = 0.008).

**Conclusions:**

Current smoking was a significant predictor of incident hypertension only in the female and middle-age populations with normal weight but not in the overweight and obesity as well as the younger and elder populations.

## Background

As well known, hypertension, as a main chronic disease, has been the greatest attributable risk factor for death worldwide [1]. Moreover, hypertension is considered as a common risk predictor for cardiovascular disease (CVD) and accounts for approximately 45% of global CVD morbidity and mortality [2, 3]. As a result, there were approximately 7 million hypertension-related deaths each year [4]. In China, the prevalence of hypertension increased from 14.5% in 1991 to 34.0% in 2012 among adult population [5, 6]. As a result, it is an urgent need to take measurements to prevent and control the development of hypertension.

Many previous studies have reported that there is an association of adiposity with hypertension [7–10]. As the major independent risk factors for hypertension, overweight and obesity account for approximately 65–78% of adult hypertension cases [8, 11–13]. On the other hand, smoking has been documented as a common risk factor for many chronic diseases, especially vascular dysfunction [14]. There was a well-established association of smoking with an increased risk of CVD [15, 16]. According to the World Health Organization (WHO) report, smoking is the leading cause of 1.69 million CVD-related deaths [17]. The positive relationship between smoking and hypertension is also well established [18–21]. However, many previous studies reported that there was a long-run effect of smoking cessation on the weight gain [22, 23]. Evenly, there were some economic literatures to report that smoking can reduce BMI [24–26]. Therefore, there might be an interaction effect between obesity and smoking on the risk of hypertension.

However, there was little known on how the interaction effect between obesity and smoking affected the incidence of hypertension. In this study, it could therefore be hypothesized that there was a significant interaction between obesity and current smoking on the incidence of hypertension among adult population. The aim of this study was to investigate how obesity modified the effect of smoking on the incidence of hypertension.

## Methods

### Study design

This study was based on the China Health and Nutrition Survey (CHNS), which is an open-cohort and national project [27]. The CHNS covers nine provinces, which are representative of the economic development and public resources in China. A multistage stratified random cluster process was employed to extract four counties from each province. A detailed description of the survey design has been published elsewhere [27, 28].

### Study population

This study covered all nine waves of the CHNS conducted from 1989 to 2011. The studied subjects should meet the following criterions: who were aged ≥18 years at baseline; who were with complete data on sex, weight, height, current smoking, and blood pressure. The subjects who were pregnant or lactating at the time of survey or with missing and implausible outlying data (e.g., weight > 300 kg or < 20 kg) would be excluded.

### Measurement and definition of indicators

Weight and height were measured by the trained healthcare workers following standardized protocols. The detailed measurements of weight and height have been described in the previous studies [8, 29]. BMI was calculated as weight in kilograms divided by the square of height in meters. Three measurements were conducted per subject for each indicator. And the average was used to analyze.

According to the WHO recommendations for Chinese people, the normal weight, overweight, and obesity were defined using the BMI cutoff values 18.5 kg/m^2^, 23.0 kg/m^2^, and 27.5 kg/m^2^ [30]. Current smokers were defined as having smoked at least 100 cigarettes or electronic cigarettes, 20 cigars, or 20 tobacco pipes and other type of tobacco (such as chewing betel quid with tobacco, dipping tobacco, and snuff tobacco) in the last 30 days preceding the survey [31, 32]. Nonsmokers were defined as they had never smoked in their lifetime. Former smokers were not mentioned in this study. Current drinking was defined as intake at least 50 g alcohol daily in the last 30 days prior to the survey [33]. Physical activity was defined as no (less than one hour per week) or yes (one or more hours per week). Under calm state, blood pressure measurements were taken with 30 s intervals between cuff inflations using standard mercury sphygmomanometers [34]. Cuff size was selected according to each participant’s arm circumference. When the first and fifth Korotkoff sounds appeared, systolic blood pressure (SBP) and diastolic blood pressure (DBP) were recorded, respectively. The same measurements were conducted for three times and the average was used. Then, hypertension was defined as SBP/DBP ≥ 140/90 mmHg, use of anti-hypertensive medications, or a self-reported diagnosis of hypertension [34, 35].

### Statistical analysis

Data are expressed as means ± standard deviations (SDs) for continuous variables and frequencies (percentages) for categorical variables. The differences of the baseline characteristics between the non-hypertension and hypertension groups were compared by *t*-tests, *chi-square* tests, and *Wilcoxon* rank sum tests for continuous normal variables, categorical variables, and ordinal variables, respectively. With hypertension as the end-event, Cox regressions were employed to examine the interaction effects between obesity and smoking as well as the associations of obesity and current smoking with the incidence of hypertension. All models adjusted for death to correct the competing risk. All Cox regression models met the proportional hazard assumption. In the adjusted models, age, sex, current drinking, physical activity, and ethnicity at baseline were adjusted. When the interaction effects appeared, the single effects of obesity and current smoking were tested using *Contrast* statement in *Phreg* procedure of SAS with Bonferroni correction for multiple comparison. All analyses were conducted using SAS 9.4 (SAS Institute Inc., Cary, NC, USA.). The two-tailed *P* ≤ 0.05 was taken as the statistical significance.

## Results

This study included 12,900 subjects, 4307 of who were hypertensive and 8593 subjects were non-hypertensive. The average of age was 38.14 years and BMI was 21.78 kg/m^2^. The median of follow-up period was 8 years. The characteristics of all subjects are shown in Table 1. The differences of all characteristics between the non-hypertension and hypertension groups were significant.

**Table 1:** The characteristics of all subjects at baseline

Characteristics	All subjects (12900)	Subgroup subjects		*t/χ*^*2*^	*P*
		Non-hypertension (8593)	Hypertension (4307)		
Age (year)^a^	38.14 ± 14.31	35.32 ± 13.52	43.75 ± 14.19	−32.320	< 0.001
BMI (kg/m^2^)^a^	21.78 ± 2.81	21.49 ± 2.70	22.34 ± 2.95	−15.810	< 0.001
Sex				90.032	< 0.001
Male	5885 (45.62)	3667 (42.67)	2218 (51.50)		
Female	7015 (54.38)	4926 (57.33)	2089 (48.50)		
Current smoking				153.265	< 0.001
No	7369 (60.00)	5126 (64.00)	2243 (52.50)		
Yes	4913 (40.00)	2884 (36.00)	2029 (47.50)		
Current drinking				121.351	< 0.001
No	7123 (57.48)	4936 (61.05)	2187 (50.78)		
Yes	5269 (42.52)	3149 (38.95)	2120 (49.22)		
Ethnicity				59.812	< 0.001
Han	10,906 (84.54)	7115 (82.80)	3791 (88.02)		
Other	1994 (15.46)	1478 (17.20)	516 (11.98)		
Physical activity				12.581	< 0.001
No	12,268 (95.10)	8131 (94.62)	4137 (96.05)		
Yes	632 (4.90)	462 (5.38)	170 (3.95)		
Obesity^b^				14.085	< 0.001
Normal	9230 (71.55)	6477 (75.38)	2753 (63.92)		
Overweight	3181 (24.66)	1883 (21.91)	1298 (30.14)		
Obesity	489 (3.79)	233 (2.71)	256 (5.94)		
Death				5.096	0.024
No	11,935 (92.52)	7982 (92.89)	3953 (91.78)		
Yes	965 (7.48)	611 (7.11)	354 (8.22)		

^a^These variables were analyzed using *t* test

^b^This variable was analyzed using *Wilcoxon* rank sum test

Table 2 shows the interaction effect between obesity and current smoking on the incidence of hypertension. When adjusting for covariates, there were interaction effects between obesity and current smoking in the total population (*P* = 0.034), females (*P* = 0.030), and those aged 50–59 years (*P* = 0.049). The further analyses were conducted to identify the single effects (Table 3). Without adjusting for covariates, overweight and obesity were associated with the incidence of hypertension despite of current smoking or not (all *P* <  0.001). While current smoking was the risk factor of hypertension among the normal weight subjects (*HR*: 1.346; *HR* 95% *CI*: 1.248–1.451; and *P* <  0.001) and the overweight subjects (*HR*: 1.235; *HR* 95% *CI*: 1.105–1.380; and *P* <  0.001) but not among the obesity subjects (*HR*: 0.917; *HR* 95% *CI*: 0.705–1.192; and *P* = 0.516). When adjusting for covariates, the effects of overweight and obesity were consistent with the crude results. But current smoking was a significant predictor of incident hypertension only among the normal weight subjects (*HR*: 1.119; *HR* 95% *CI*: 1.013–1.236; and *P* = 0.027).

**Table 2:** The interaction effect between obesity and current smoking on the incidence of hypertension

The interaction terms	Crude		Adjusted^a^	
	*β*	*P*	*β*	*P*
**Total population**				
obesity ×current smoking	− 0.172	0.001	−0.113	0.034
**Males**				
obesity ×current smoking	− 0.219	0.007	− 0.089	0.273
**Females**				
obesity ×current smoking	−0.406	0.002	−0.280	0.030
**Aged 18–29 years**				
obesity ×current smoking	−0.084	0.551	−0.095	0.499
**Aged 30–49 years**				
obesity ×current smoking	−0.110	0.180	−0.111	0.180
**Aged 50–59 years**				
obesity ×current smoking	−0.216	0.034	−0.202	0.049
**Aged 60~93 years**				
obesity ×current smoking	0.115	0.418	0.120	0.398

^a^In adjusted model, age, gender, current drinking, ethnicity, physical activity, and death were adjusted

**Table 3:** The single effects of obesity and current smoking on the incidence of hypertension in total population

	Crude			Adjusted^a^		
	*HR*	*HR* 95% *CI*	*P*	*HR*	*HR* 95% *CI*	*P*
Overweight and nonsmoking	2.480	1.870–3.288	< 0.001	1.990	1.500–2.640	< 0.001
Obesity and nonsmoking	3.700	3.148–4.349	< 0.001	2.872	2.441–3.378	< 0.001
Overweight and current smoking	2.276	1.642–3.154	< 0.001	1.878	1.354–2.604	< 0.001
Obesity and current smoking	3.396	2.792–4.131	< 0.001	2.710	2.227–3.299	< 0.001
Current smoking and normal weight	1.346	1.248–1.451	< 0.001	1.119	1.013–1.236	0.027
Current smoking and overweight	1.235	1.105–1.380	< 0.001	1.056	0.930–1.198	0.400
Current smoking and obesity	0.917	0.705–1.192	0.516	0.893	0.683–1.167	0.406

^a^In adjusted model, age, sex, current drinking, ethnicity, physical activity, and death were adjusted

In Cox regression models stratified by sex, there was no interaction effect between obesity and current smoking in males. Therefore, the single effects were displayed only in females (Table 4). As adjusting for covariates, overweight and obesity were the significant predictors of hypertension despite of current smoking or not (*HR*: 2.726, 3.074, 2.377, and 2.681; *HR* 95% *CI*: 1.541–4.821, 2.570–3.678, 1.161–4.867, and 1.909–3.764; and *P* <  0.001, < 0.001, 0.018, and <  0.001, respectively). However, current smoking was associated with hypertension only in the normal weight subjects (*HR*: 1.274; *HR* 95% *CI*: 1.143–1.415; and *P* = 0.040), but not in the overweight (*P* = 0.851) and obesity subjects (*P* = 0.090).

**Table 4:** The single effects of obesity and current smoking on the incidence of hypertension in females

	Crude			Adjusted^a^		
	*HR*	*HR* 95% *CI*	*P*	*HR*	*HR* 95% *CI*	*P*
Overweight and nonsmoking	3.345	1.893–5.912	< 0.001	2.726	1.541–4.821	< 0.001
Obesity and nonsmoking	3.827	3.203–4.571	< 0.001	3.074	2.570–3.678	< 0.001
Overweight and current smoking	2.623	1.282–5.364	0.008	2.377	1.161–4.867	0.018
Obesity and current smoking	3.000	2.139–4.207	< 0.001	2.681	1.909–3.764	< 0.001
Current smoking and normal weight	1.864	1.563–2.225	< 0.001	1.274	1.143–1.415	0.040
Current smoking and overweight	1.462	1.147–1.862	0.002	1.024	0.802–1.405	0.851
Current smoking and obesity	0.795	0.474–1.334	0.386	0.639	0.381–1.072	0.090

^a^In adjusted model, age, current drinking, ethnicity, physical activity, and death were adjusted

**Fig. 1:**
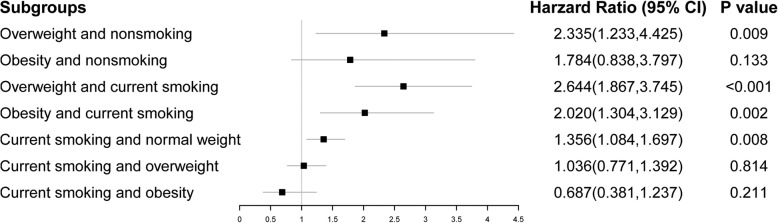
The single effects of obesity and current smoking on the incidence of hypertension in the 50–59 years group

The single effects of obesity and current smoking on the incidence of hypertension in the 50–59 years group are displayed in Fig. 1. Overweight and obesity were the predictors of hypertension except in the obesity subjects with nonsmoking (*HR*: 1.784; *HR* 95% *CI*: 0.838–3.797; and *P* = 0.133). And current smoking was a predictor of hypertension only among the normal weight subjects (*HR*: 1.356; *HR* 95% *CI*: 1.084–1.697; and *P* = 0.008).

## Discussion

By now, there were few studies to investigate the interaction effect between obesity and current smoking on incident hypertension. This study was based on the CHNS, a 22-year national cohort study, and attempted to examine the interaction effect. The results implied that there were significant interaction effects between obesity and current smoking in females and the 50–59 years population. Despite of current smoking or not, obesity was a significant predictor of incident hypertension. However, current smoking was associated with hypertension only in the female and middle-aged subjects with the normal weight. When subjects were overweight or obesity, current smoking was ineffective on the incidence of hypertension.

Although previous studies reported that acute effect of smoking could temporarily raise blood pressure [17, 36, 37], several population-based studies have shown that there was no or a negative relationship between chronic smoking and hypertension [38–40]. Perhaps obesity, as a confounding factor, covered the effect of smoking. Notably, the prevalence of smoking and hypertension have been decreasing, but accompanied with a simultaneous increase in obesity [41]. As a result, the effect of smoking on hypertension might reduce or disappear but the effect of obesity on hypertension would be more significant. However, the mechanism of the interaction effect between obesity and smoking is unknown and should be further investigated in the future study.

This study showed that current smoking was a significant risk factor for hypertension in females but not in males. It was consistent with the previous studies [19, 42]. A season might be the different characteristics between males and females. The characteristics of male smokers were smoking for a long time and heavy smoking but relatively lower proportion of smokers and hypertension in females [43].

### Limitations and strengths

This study was based on a 22-year follow-up national population-based cohort study. As a result, the conclusion would be more comprehensive and convictive. Furthermore, it was the first study to examine the interaction effect between obesity and current smoking on the incidence of hypertension. The results would provide a new insight for prevention and control of hypertension. However, the limitations of this study should be stated. First, in this study, smoking status was obtained according to the self-reported questionnaire. There might be misclassification error and recall bias. Second, since the information of former smoking was unavailable, the former smoking was not considered in this study. Third, since the sodium intake is not available due to the limitation of the CHNS and the family history of hypertension is not collected in the CHNS, the diet intake and the family history of hypertension were not adjusted in this study.

## Conclusions

Overweight and obesity were associated with the higher incident hypertension both in the smokers and the non-smokers. However, current smoking was a significant predictor of the incidence of hypertension only in the female and middle-aged populations with the normal weight but not in the overweight and obesity as well as the younger and elder populations. Therefore, different public health interventional measurements should be made for different populations.

## Data Availability

The datasets analyzed during the current study are available in the China Health and Nutrition Survey (CHNS) on the site: https://www.cpc.unc.edu/projects/china.

## Abbreviations

CHNS: China Health and Nutrition Survey
CVD: Cardiovascular disease
SDs: Standard deviations
WHO: World Health Organization
